# Exploring the Gut-Brain Connection: Are Probiotics the Next Frontier in Alzheimer’s Disease Treatment?

**DOI:** 10.1007/s12602-026-10927-w

**Published:** 2026-03-04

**Authors:** Eduarda Behenck Medeiros, Adrielly Vargas Lidio, Gabriel Casagrande Zabot, Gabriela Piovesan Fenilli, Gustavo de Bem Silveira, Gabriela Serafim Keller, Flávio Rigoberto Arriagata Carrion, Fernanda Guarino De Felice, Bruno Kluwe-Schiavon, Consuelo Walls-Bass, Tatiana Barichello, Josiane Budni

**Affiliations:** 1 Laboratory of Experimental Neurology, Graduate Program in Health Sciences (PPGCS), University of Southern Santa Catarina, UNESC, Av. Universitária, 1105 - Universitário, Criciúma, SC 88806-001 Brazil; 2 D’Or Institute for Research and Education (IDOR), Rio de Janeiro, Brazil; 3 Faculdad de La Salud, Universidad del Alba, Region Metropolitana, Av. Ejército Libertador n° 171-177, Santiago, Chile; 4 Institute of Medical Biochemistry Leopoldo de Meis, Federal University of Rio de Janeiro, Rio de Janeiro, Brazil; 5 Center for Neuroscience Studies, Department of Biomedical and Molecular Sciences, Department of Psychiatry, Queen’s University, Kingston, ON Canada; 6 Faillace Department of Psychiatry and Behavioral Sciences, McGovern Medical School, University of Texas Health Science Center at Houston, 1941, East Road, Houston, TX 77054 USA

**Keywords:** Alzheimer's disease, Gut, Microbiota, Probiotics

## Abstract

Alzheimer’s disease (AD) is the most common form of dementia, historically considered exclusively as a neurological condition treated primarily with cholinergic and glutamatergic inhibitors. Recent evidence highlights the significant influence of peripheral systems, particularly the gut-brain axis, on AD pathology. The gut microbiota plays a critical role in both physiological and psychological functions, and its interactions may impact the integrity of the blood-brain barrier, potentially contributing to neurodegenerative processes. Emerging therapeutic strategies targeting the gut microbiome, including both pharmacological and non-pharmacological approaches such as probiotics and prebiotics, offer promising avenues for intervention. This review synthesizes current literature and illustrative data to elucidate the connections among the gut-brain axis, neuroinflammation, and neuroprotection, and their implications for the pathogenesis and potential treatment of AD.

## Alzheimer Disease

Alzheimer’s disease (AD) is the most prevalent form of dementia, being responsible for up to 80% of all dementia cases. Dementia commonly affects people over 65 years of age, primarily women. Studies show that women account for 2/3 of dementia diagnoses, representing 12% of dementia cases, and men represent only 10% in the United States (US) [1, 2]. The primary pathophysiology comprises amyloid-β (Aβ) accumulation, a hydrophobic peptide that aggregates into amyloid plaques, and tau protein hyperphosphorylation, which aggregates intracellularly into neurofibrillary tangles [3]. Beyond these, other alterations have been studied, including epigenetics, inflammation, and the microbiota-gut-brain axis, to gain a deeper understanding of the disease, which could lead to the development of alternative therapeutic strategies.

The function of the amyloid precursor protein (APP) remains unknown. It is a transmembrane protein in neurons that is usually cleaved to form Aꞵ_16−17_, a hydrophilic peptide. For numerous reasons, such as genetics, aging, or external factors, a different cleavage of APP becomes more prevalent, leading to the formation of the hydrophobic and toxic peptide Aβ_40−42_. These aggregate outside of the neuron, initiating an inflammatory process that blocks ionic channels, increases reactive oxygen species (ROS) production, alters calcium homeostasis, and reduces energetic metabolism, ultimately leading to neuronal death [3].

AD progresses gradually, beginning with a preclinical phase in which the pathological hallmarks are present, but no objectively verified cognitive symptoms are observed. This preclinical phase, named presymptomatic, can last for several years or even decades. It is followed by a prodromal phase, during which cognitive symptoms begin to emerge and worsen in severity, finally resulting in dementia. In this advanced stage, patients lose the ability to perform activities of daily living independently. Generally, diagnosis is performed at this stage, which is later [4–6].

Besides exploring the pathogenesis of AD and new treatments, the need for early diagnosis is also growing. Recently, the use of plasma biomarkers has facilitated early-stage diagnosis. When detected early, the prognosis for improvement with new therapies tends to be higher [4, 5, 7].

Among the numerous biomarkers are assays to measure Aβ peptide and tau protein in cerebrospinal fluid (CSF), positron emission tomography (PET) imaging for detecting AD proteinopathies, volumetric magnetic resonance imaging (MRI), and accurate blood-based biomarkers that replicate CSF findings. Currently available biomarkers enable precise differential diagnosis of AD and provide essential staging and prognostic information [6, 8].

**Fig. 1:**
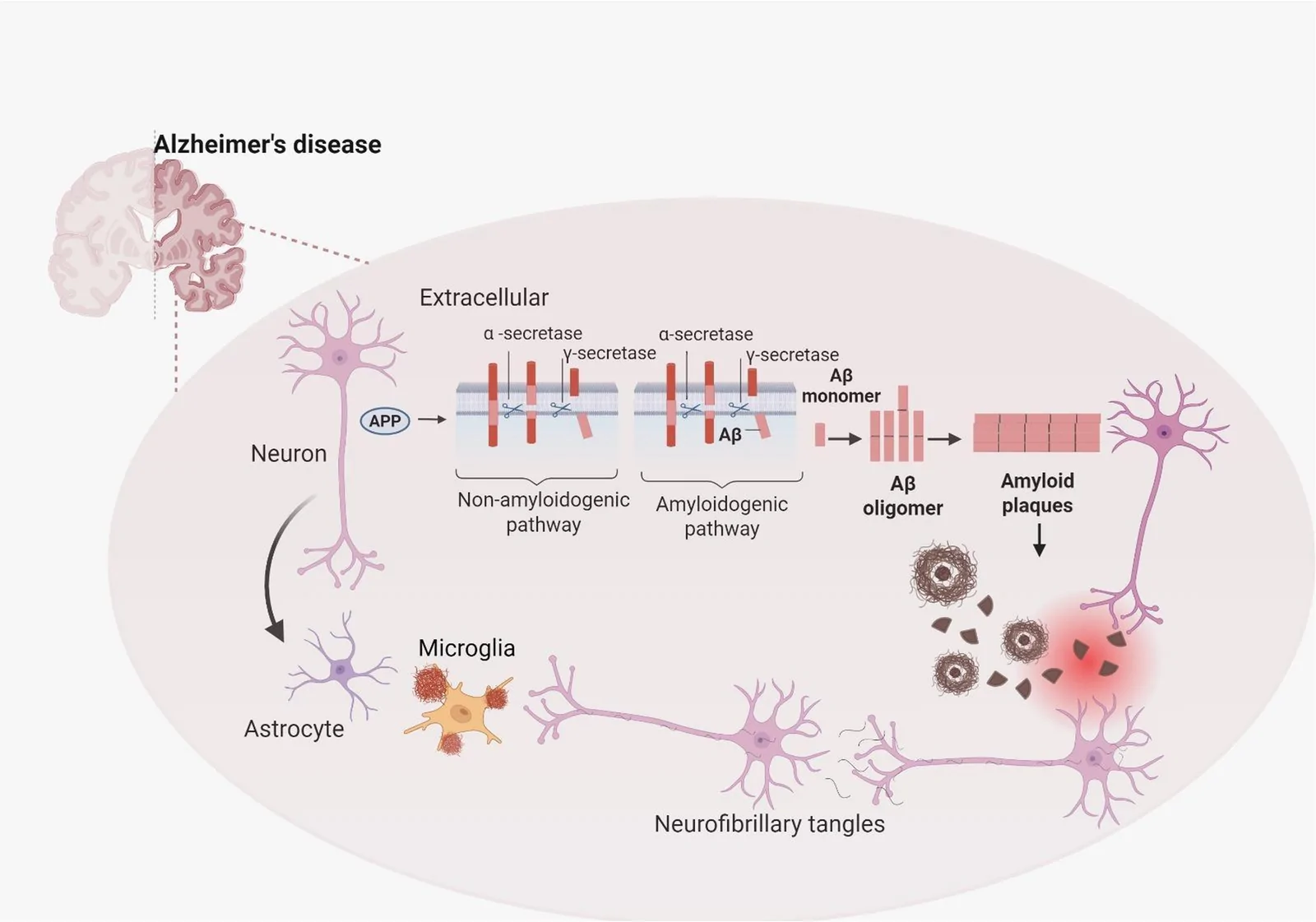
Pathophysiological cascade of Alzheimer’s disease. In the amyloidogenic pathway, the APP is sequentially cleaved by β-secretase and γ-secretase, resulting in the production of Aβ peptides. These peptides can aggregate into insoluble Aβ oligomers, ultimately leading to extracellular Aβ plaques that are toxic to neurons. In contrast, the non-amyloidogenic pathway involves cleavage by α-secretase and γ-secretase, which precludes Aβ formation. Aβ oligomers activate astrocytes and microglia, triggering neuroinflammation and contributing to synaptic dysfunction and neuronal loss. These pathological processes are associated with the development of neurofibrillary tangles and cognitive decline in AD. Figure created with the assistance of BioRender (https://biorender.com).

Concomitant with the erroneous cleavage of Aβ, hyperphosphorylation of the tau protein is another characteristic pathophysiology of AD. Tau stabilizes neurons’ microtubules. However, upon contact with protein kinases, which are abundant due to the inflammatory process caused by Aβ oligomers, it becomes hyperphosphorylated, dissociates from microtubules, and aggregates intracellularly, forming neurofibrillary tangles [9]. With its dissociation from microtubules, communication between neurons gradually deteriorates, signaling processes are compromised, and the cell undergoes apoptosis [3]. In Fig. 1, we explain the AD pathophysiology.

Although the disease was discovered more than 100 years ago, and several pathological pathways have been proposed, its pathogenesis remains incompletely elucidated. However, there is recurring evidence of a relationship between gut microbiota and AD. Gut microbiota interact with the brain in various ways, including regulating neuroinflammation, modulating Aβ production and clearance, influencing tau phosphorylation, controlling neurotransmitters, and managing oxidative stress [10, 11]. With this in mind, new studies have focused on identifying the relationship between probiotics and neurodegenerative diseases, particularly neuroinflammation, which appears to be the most significant association.

## Neuroinflammation

Neuroinflammation is an inflammatory response in the central nervous system (CNS) [12, 13]. The causes of neuroinflammation are vast, including infectious diseases, aging, toxins, systemic inflammation, and trauma [14]. Mediated mainly by microglia, neuroinflammation consists of increased production of pro-inflammatory cytokines, chemokines, prostaglandins, ROS, and nitric oxide [14]. Not only are pro-inflammatory mediators increased in a neuroinflammatory board. Anti-inflammatory cytokines, such as interleukin (IL) IL-4 and IL-10, have also been observed to be increased in AD animal models [15]. These anti-inflammatory cytokines are likely elevated to help control the inflammatory response. However, in the case of AD and other dementias, neuroinflammation tends to become a chronic process and cannot be controlled [16].

In the early stages of AD, Aβ is recognized by microglial receptors, such as TREM-2, which facilitates Aβ phagocytosis. However, TREM-2 activation leads to the production of pro-inflammatory cytokines and ROS [17]. In the long run, increased production of pro-inflammatory cytokines will impair microglial phagocytic function and promote the progression of AD. IL-1β could increase tau phosphorylation, as demonstrated by a study using IL-1β blockers, which suppressed the activation of cdk5/p25, GSK-3β, and p38-MAPK [18]. TNF-α production is also increased in AD patients. Due to this cytokine’s ability to interfere with glutamatergic receptors in the synaptic cleft, it could be one of the factors responsible for glutamatergic neurotoxicity and neuronal death [19, 20].

The increase in inflammation can be detected in both the cerebrospinal fluid (CSF) and the blood of AD patients [21, 22]. Patients with amyloid brain lesions and cognitive impairment show an increase in pro-inflammatory chemokines when compared to healthy individuals [23]. Furthermore, studies have shown that the Aβ peptide can activate pattern recognition receptors (PRRs) and consequently, the innate immune system [24, 25]. Immune activation triggers inflammation through TREM-2, as mentioned earlier, and through others such as NLR and NLRP3g, ASC, CD33, CD22, and CD68 [26, 27]. Thus, the accumulation of Aβ leads to its phagocytosis by microglia, which can result in microglial reactivity (microgliosis), the release of pro-inflammatory cytokines, hyperphosphorylation of tau, synaptic dysfunction, neuronal loss, and cognitive impairment. Dysfunctional microglia are less capable of phagocytosing Aβ peptide within compact plaques and are less able to inhibit the increase of plaques and tau near the plaques, resulting in neuronal damage [28–30].

During the early stages of AD, microglia appear to have a positive impact on disease progression, as they can phagocytose Aβ peptide. After the disease becomes chronic, however, microglia appear to lose their efficiency in clearing Aβ peptide, and may induce increased production of apolipoprotein E4 (APOE4) in a TREM-2-dependent manner, leading to more Aβ plaque formation [31].

TREM-2 is a transmembrane receptor expressed in microglia. It has many ligands, including lipids and APOs [31]. TREM-2 is one of the receptors activated by Aβ and is also an important factor for the phagocytosis of Aβ peptide. As described by Fruhwürth et al. (2024), microglia may also be involved in plaque seeding, especially when Aβ peptide is overabundant. At later stages of AD, microglia are believed to contribute to plaque compaction and barrier formation, which potentially prevents neuritic dystrophy. Due to their close association with Aβ plaques, microglia have been suggested to be causally involved in various stages of Aβ plaque formation, including plaque seeding, compaction, and isolation [32].

After phagocytosis by microglia, via Toll-like receptors (TLR-2, −4, and − 6), Aβ peptide activates the NLRP3 inflammasome, leading to activation of caspase-1 and maturation and release of IL-1β [33]. The NLRP3 inflammasome is activated in brains with AD and mild cognitive impairment (MCI) and APP/PS1 mice [34, 35]. Genetic deletion of NLRP3 and caspase-1 results in decreased Aβ peptide deposition, reduced IL-1β release from microglia, and improved cognitive performance in APP/PS1 mice [36]. Glial markers IBA and CD68 can indicate the phagocytic state of microglia, contributing to the understanding of this inflammasome pathway [37].

In older individuals, a chronic state of inflammation known as *inflammaging* exists. This inflammation affects the entire organism, including the intestinal mucosa, where an imbalance in the intestinal microbiota can be observed, leading to reduced diversity and stability [38]. Alterations in the gut microbiota can trigger intestinal inflammation, which, in turn, can stimulate neuropathology, such as AD, in the gut. It is hypothesized that this inflammation, through the vagus nerve, can be transmitted to the CNS, triggering AD [27].

Neuroinflammation, characterized by reactive glial cells (astrocytes and microglia), is also an essential feature of AD. Usually, microglia help maintain synapses in the CNS, but in the presence of pathogens or inflammatory stimuli, they acquire a reactive, amyloid form [39]. In their homeostatic state, microglia express all TLRs at a low basal level; however, upon reactivity, their expression increases, enhancing their ability to recognize and be activated by PAMPs and DAMPs, generating inflammation and thus exerting their immunosurveillance function in the brain [40]. A physiological imbalance in the CNS induces its activation, thereby increasing phagocytic capacity and production of inflammatory cytokines. This transformation is evident in aging and AD neurodegeneration, where microglia and astrocytes exhibit reactivity to Aβ peptides and form Aβ plaques and neurofibrillary tangles in the CNS [41].

Neuroinflammation in the CNS ultimately results in a vicious cycle in which Aβ peptide stimulates microglia via pathways such as the nuclear factor kappa B (NF-κB) and MAPK (mitogen-activated protein kinase) pathways, leading to the release of cytokines such as TNF-alpha, IL-1β, and IL-6. The established exacerbated inflammatory state contributes to the dysfunction and death of CNS cells. In addition to inflammation, biochemical evidence has demonstrated that the mitochondrial transport chain is affected in AD. Specifically, the enzyme cytochrome c oxidase (complex IV) of the respiratory chain is involved in this pathology [42].

## Microbiota-Gut-Brain-Axis

The gut microbiota, also known as intestinal flora, comprises various microorganisms that reside in the gastrointestinal tract (GIT). The colonization process starts at the beginning of life. It persists throughout the years because microbiota are individual and have unique characteristics for each living being, which are conditioned by genetic and epigenetic factors [43, 44]. Everyone has a particular group of microorganisms, and this number is equivalent to the number of human cells. These microorganisms collectively contain more than 100 times the number of human genes [45]. The microbiota of the adult can weigh up to 1 kg, and most of it is found on the colon [46–48].

In adult individuals, the phylum *Firmicutes* (51%) prevails, comprising the groups *Clostridium coccoides* and *C. leptum*, as well as the genus *Lactobacillus*. The *phylum Bacteroidetes* (48%) is also represented, including the genera *Bacteroides* and *Prevotella*. The less populous phyla constitute the remainder of the population, including *Proteobacteria*,* Fusobacteria*,* Spirochaetae*,* Verrucomicrobia*,* Lentisphaerae*, and *Actinobacteria*, the latter of which includes the genus *Bifidobacteria* [49]. GIT is relatively balanced in terms of quantity and diversity of these microorganisms, and changes in these factors are associated with the development of multiple diseases [50].

In addition to presenting distinct flora, the microbiota can exhibit great diversity by sex. A study by Ma et al. (2020) showed that in aged mice, males and females presented distinct changes. Male mice showed a significant reduction in the phylum *Actinobacteria*, and females showed an increase in the phylum *Firmicutes* [51]. These divergences can be attributed to hormonal changes, particularly those of estrogen and testosterone [52].

Besides sex changes, dietary habits also have an influence. Diet plays a crucial role in gut microbiota composition. The Western diet, typically rich in saturated fats, refined sugars, and ultra-processed foods, promotes gut dysbiosis and a chronic state of inflammation [53]. This pro-inflammatory state contributes to systemic metabolic disturbances and neuroinflammatory processes that may accelerate the progression of AD pathophysiology. In contrast, adherence to the Mediterranean diet, characterized by a high intake of fruits, vegetables, legumes, whole grains, olive oil, and omega-3 fatty acids, supports a more diverse and balanced gut microbiome [54, 55]. This diet enhances gut barrier integrity, reduces oxidative stress and inflammation, and exerts neuroprotective effects [56].

The intestine functions extend beyond being an absorptive tube that transports, digests, absorbs, and excretes nutrients and fluids; it is also associated with the immune, endocrine, and neurological systems [57]. The intestinal mucosal lymphatic tissue is considered the most significant immunological organ, accounting for 70–80% of the human immune system [50, 58, 59].

Changes in the gut microbial flora can occur throughout life due to various external factors, including poor eating habits, nutrient-deficient diets, consumption of ultra-processed foods, excessive energy intake, stress, and the overuse of antibiotics [42]. These changes can lead to the emergence of certain diseases, such as obesity, inflammatory bowel disease, type 2 diabetes, and even neurodegenerative diseases [60–63]. Additionally, Claesson et al. (2012) demonstrated that elderly individuals, whether institutionalized or community-dwelling, exhibited differences in intestinal flora, with institutionalized individuals showing a decrease in intestinal microbiota, suggesting an environmental influence [64].

In addition to the factors already mentioned that can influence intestinal microbiota, we are still trying to understand whether factors beyond aging are needed to cause changes in microbiota, without relating them to other external factors [61]. With all these changes, the gut-brain axis has become a promising target for understanding the physiopathology of psychological disorders and cognitive impairment [65–67].

Changes in the microbiota may influence neurotransmitters and affect communication along the gut-brain axis [63]. The gut-brain axis connection is complex and bidirectional, involving immune, endocrine, and neural pathways. Communication between the intestine and the CNS occurs via the vagus nerve, which connects intestinal neurons to the CNS. The vagus nerve connects to the spinal cord, the autonomic nervous system (ANS), and the efferent and afferent fibers of the brainstem, allowing the brainstem to control various intestinal functions [44, 48, 63]. In addition to the vagus nerve, intestinal chemicals can cross the gut epithelial and vascular barriers and reach the CNS via the blood-brain barrier (BBB). BBB and the intestinal mucosa allow the passage of specific molecules, such as cytokines, that can influence both the brain and the intestine. Some studies have shown that the gut–brain neuronal signaling axis is initiated by the secretion of gut hormones [68, 69].

**Fig. 2:**
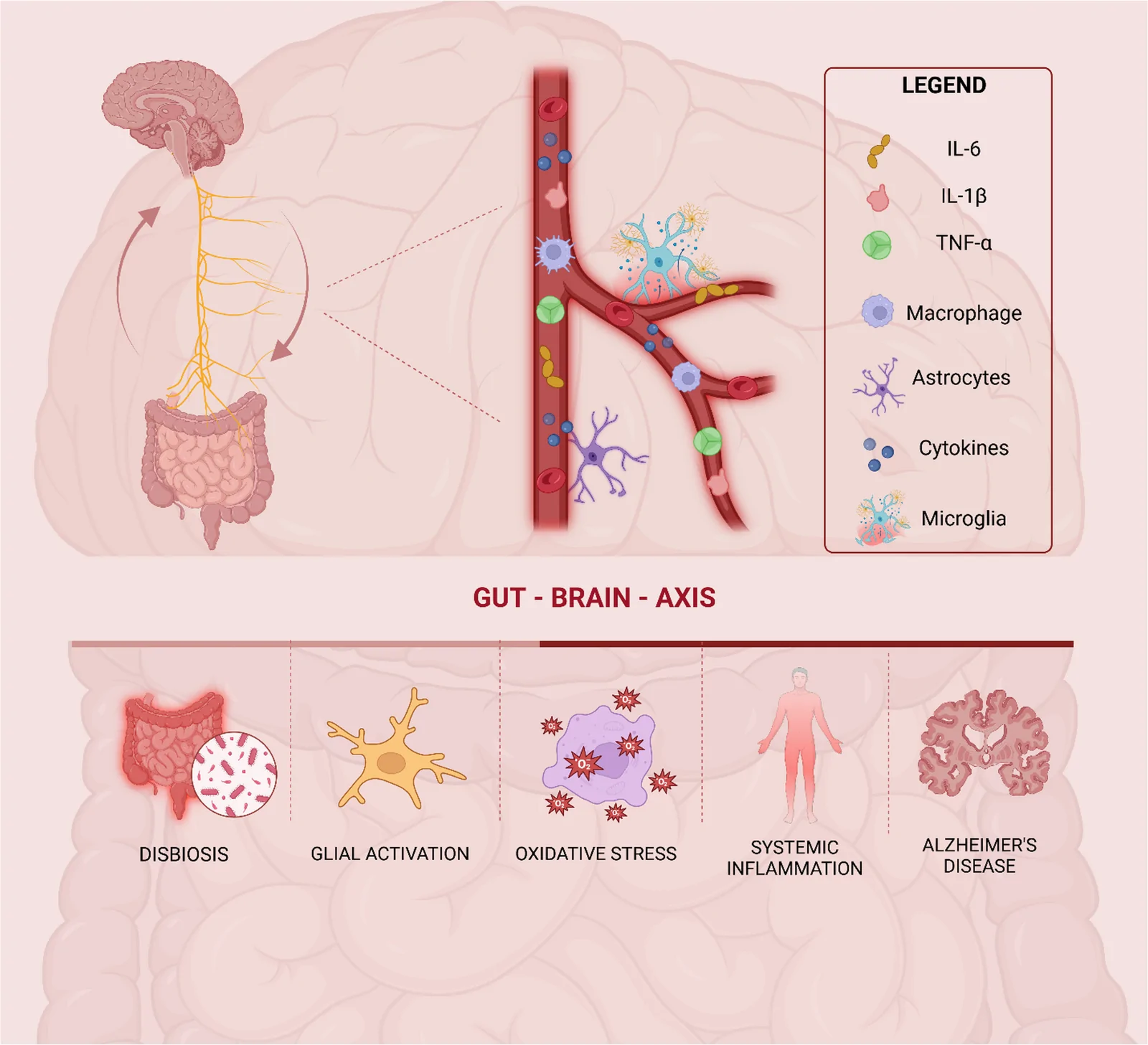
Connection between gut and brain through vagus nervus and the neuroinflammation, glial activation, and systemic processes in the gut-brain axis. Gut dysbiosis can increase intestinal barrier permeability, leading to the release of microbial metabolites and pro-inflammatory cytokines, such as IL-6, IL-1β, and TNF-α, into the systemic circulation. These inflammatory mediators can cross the BBB or affect it, promoting glial activation in the CNS, including astrocytes and microglia. This process triggers oxidative stress and neuroinflammation, contributing to the development and progression of AD. The vagus nerve serves as a bidirectional communication pathway between the gut and the brain, underscoring the significant influence of the gut microbiota on brain health. Figure created with the assistance of BioRender (https://biorender.com*)*

Microbiota play a crucial role in regulating disease. In a healthy organism, microorganisms regulate the digestive pH and create a protective barrier against infectious agents. It is present in our body since birth and can vary according to age [62, 63]. In cases of intestinal inflammation, microbiota releases more pro-inflammatory cytokines that could potentially harm the brain [62]. Although gut microbes do not age, comorbidities, dysbiosis, and inflammation associated with the gut microbiota increase with age. It was observed that in patients with AD, the microbiota has lower levels of bacteria and decreased levels of butyrate, leaving the intestinal mucosa more permeable and potentially leading to increased brain inflammation and, consequently, cognitive loss and diseases such as AD [43, 63, 70]. In Fig. 2. we explain the primary correlation between neuroinflammation and the gut-brain axis.

## Gut-Brain-Axis and Alzheimer’s Disease

The vagus nerve primarily mediates neuronal communication between the gut and the brain. This communication pathway is guided by the enteric nervous system, in which dysbiosis, combined with intestinal inflammation, is a key trigger of its activation. Imbalance in this pathway can occur due to misfolded proteins, with evidence that tau pathology can propagate through efferent pathways of the vagus nerve in AD models, and ascending inflammatory signals activate microglia. Thus, neuroimmune dysfunction and vagus nerve communication provide the neuronal mechanisms by which the gut directly contributes to persistent neuroinflammation and the progression of neurofibrillary and amyloid pathology in AD [71, 72].

The first correlation between AD and gut microbiota was reported in the context of inflammation [73]. Changes in the microbiota occur when intestinal permeability increases, leading to a rise in pro-inflammatory cytokines [63, 66, 74]. Neuroinflammation is the most widely accepted mechanism linking the gut microbiome to AD [74]. In addition, a study by Cattaneo et al. (2017) demonstrated that older adults with AD have higher levels of pro-inflammatory bacteria and lower levels of anti-inflammatory bacteria than healthy older adults, corroborating the hypothesis [75].

In addition, bacteria such as *Escherichia coli*, *Salmonella enterica*, *Bacillus subtilis*, *Mycobacterium tuberculosis*, and *Staphylococcus aureus* produce extracellular amyloid fibers. These amyloid proteins differ from human proteins in their primary structure but have similarities in their tertiary structure. This similarity could activate the immune system, predisposing the individual’s brain to the formation of amyloid peptide. These studies showed an association between gut microbiota composition and brain amyloid levels [63, 76, 77]. Besides this, a gut microbiome with more *Fusicatenibacter*, *Blautia*, and *Dorea* or *Firmicutes*, *Proteobacteria*, and *Bacteroidetes* is present in patients with AD or MCI, which can be associated with lower scores in cognitive tests and an increase in pro-inflammatory mediators, as IL-6, CXCL2, NLRP3, and IL-1β [75, 77, 78].

Corroborating these hypotheses, a study by Sun et al. (2020) investigated the effects of injecting Aβ_1–42_ into the gastric wall [79]. One year later, the researchers observed Aβ_1–42_ oligomers in the vagus nerve and the brains of the mice. In addition to Aβ_1–42_, the animals exhibited cognitive impairment, corroborating the gut-brain-AD hypothesis. Microbiome dysbiosis was shown to have adverse effects on brain function and host behavior, suggesting its potential role in the development of AD. It is thought to be involved in AD through alterations in at least five distinct pathogenic processes. These include Aβ deposits, neuroinflammation, oxidative stress, and glial activation. Individuals with gut microbiome dysbiosis, poor diet, and sedentary lifestyle increase inflammation and the risk of developing AD [80–82].

The inflammatory process related to dysbiosis and the gut-brain axis may also be linked to and activated by mitochondrial dysfunction. Short-chain fatty acids (SCFAs) present in the intestine exert effects on mitochondria, regulating mitochondrial function, supporting mitophagy and redox balance, and favoring the maintenance of bioenergetic function. However, when dysbiosis is present, this regulation can be impaired. Intestinal changes, such as increased lipopolysaccharide (LPS) and intestinal permeability triggered by dysbiosis, can activate immune responses, generate oxidative stress, directly compromise mitochondrial bioenergetics, and feedback into the cycle of dysregulation and inflammation [83, 84].

The main explanation for the relationship between dysbiosis and AD is the bidirectional connection between the gut and the brain. However, to date, the best reason for this causal relationship is that dysbiosis triggers changes in the intestinal flora, such as increased LPS levels and decreased levels of *Lactobacillus* and *Bacteroides*. These bacteria are necessary for SCFA production, which, in turn, contributes to the integrity of the intestinal mucosa. The permeability of the intestinal mucosa and the presence of LPS increased, in turn leading to the breakdown of the BBB and to neuroinflammation in the CNS through the activation of glial cells such as astrocytes and microglia [63, 85, 86] (Fig. 2).

Additionally, recent evidence has expanded the focus beyond gut microbiota to encompass the role of oral microbiota in systemic and neuroinflammatory processes. When the oral microbiota undergoes modification, leading to increased levels of bacteria such as *Porphyromonas* *gingivalis* and *Fusobacterium nucleatum*, often triggered by periodontal disease, dysbiosis also occurs [87]. *Porphyromonas gingivalis*, a key pathogen in periodontitis, disrupts the oral microbial balance [88]. Dental caries and periodontal disease are significant sources of chronic oral inflammation, altering the composition of the oral microbiome [89, 90].

Oral dysbiosis can release endotoxins, such as LPS, which can cause systemic inflammation by increasing permeability of the oral mucosa or the bloodstream, thereby activating the immune system. Another way this cascade can be triggered is through saliva. We swallow large amounts of saliva daily, allowing oral bacteria to reach the intestine. When the immune system is activated, it initiates the cytokine cascade, releasing IL-1β, IL-6, and TNF-α, which contribute to neuroinflammation [88].

The oral microbiota, as the primary instigator of periodontitis, represents a potential mechanistic link between oral infections and increased gut dysbiosis. We discussed in the previous session that gut dysbiosis and its consequences contribute to the pathogenesis of AD, driving disease progression via microbial translocation and inflammation [57, 91, 92].

Despite several hypotheses for AD, such as the Aβ hypothesis, tau protein, and inflammation, dysbiosis has emerged as a probable mechanism that supports these hypotheses [80, 82].

## Current Treatments

Although it is a disease that has been known for over 100 years, only recently has the Food and Drug Administration (FDA) approved a treatment that directly targets the pathology of AD. Traditionally, care such as physical activity and cognitive stimulation activities are used, as epidemiological data suggest that physical activity and years of education are essential protective factors against AD [93–95]. Alongside these care measures, it is common to use an acetylcholinesterase inhibitor (AChEI) and/or memantine, which is a non-competitive N-methyl-D-aspartate (NMDA) receptor inhibitor [96].

Until recently, there was no pharmacological therapy directly targeting AD, so the focus of the treatment would be on preserving quality of life through symptomatic therapies. Among available drugs, AChEIs such as donepezil, galantamine, and rivastigmine are widely used to alleviate symptoms by increasing acetylcholine availability by inhibiting its degradation in the synaptic cleft. Their side effects are tolerable, but the drug should be administered with caution in patients with cardiac pathologies due to the risk of bradyarrhythmia [97].

To complement the treatment, there is also memantine, the only NMDA receptor antagonist approved as a treatment for AD. It is a drug with moderate affinity, a non-competitive, voltage-dependent NMDA receptor antagonist. Used as an alternative and/or complementary treatment, its main objective is to prevent glutamatergic neurotoxicity caused by the disease [97, 98]. It is used in moderate-to-advanced AD, both alone and in combination with an AChEI. Its combination therapy is more effective than a single AChEI [96, 98].

In 2021, the US FDA approved aducanumab for the treatment of AD. Aducanumab is a monoclonal antibody targeting the N-terminal region of the Aβ peptide, designed to engage the patient’s immune system to combat one of the primary pathologies of AD [99]. It has a few contraindications, but its main side effect is called “amyloid-related imaging abnormalities,” also known as ARIA, which is a classification for brain vascular accidents due to treatment [100]. It is an approved treatment through an accelerated pathway, due to the expectation of treatment efficacy (reduction ofAβ plaques); however, if this does not occur, the FDA may revoke the drug’s license [99].

## Prebiotics and Probiotics

Despite the constant effort to develop pharmaceutical treatment for AD, much attention has been given to other options for managing the disease lately. Moreover, prevention and management approaches might be a more realistic way of dealing with the disease before a drug treatment is a viable solution. And the gut-brain axis has been showing promising results in its relationship with neurodegenerative diseases, such as AD [101–105].

New experiments are emerging with promising results in animal models and in humans after probiotic treatment [104, 105]. Probiotics are living microorganisms that, when consumed in adequate amounts, confer health benefits to the host. They can exert various effects in the body, including inhibiting harmful microbes, modulating the immune system, supporting gut health, balancing the body’s pH, regulating immunity, controlling inflammation, and boosting the brain-derived neurotrophic factor (BDNF) [106]. Meanwhile, prebiotics are nutrients for these bacteria (mostly fiber) [107].

The bacteria involved in the gut microbiome vary from person to person. Still, it has been demonstrated that patients with AD have a prevalence of pro-inflammatory bacteria, such as *Bacteroidetes*, and a reduction in anti-inflammatory bacteria, such as *Bifidobacteria* [102, 108]. It is not yet clear whether this shift in gut microbiota comes before or after disease development. However, it does influence systemic and CNS inflammation, affecting the individual’s cognition and possibly even accelerating disease progression [63].

**Fig. 3:**
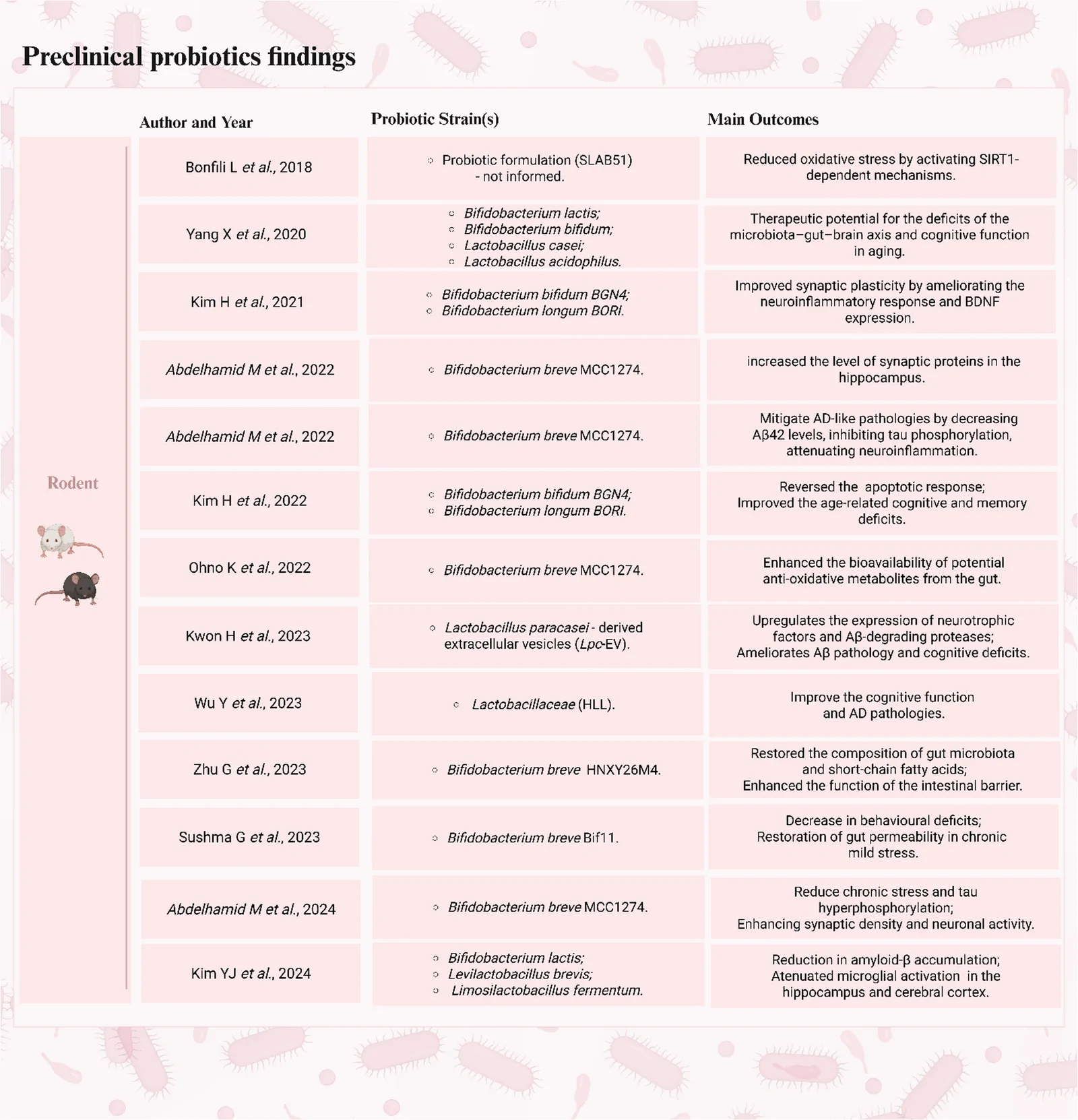
Preclinical findings on the use of probiotics in animal models. The table summarizes key studies conducted in rodent models, highlighting authors, the probiotic strains administered, and the main outcomes reported. Results include improvements in cognitive function, reduced oxidative stress, modulation of gut microbiota composition, enhanced synaptic plasticity, reduced AD–related pathological markers, and strengthened intestinal barrier integrity. Figure created with the assistance of BioRender (https://biorender.com*).*

To evaluate the influence of gut microbiota, many studies have used prebiotics and probiotics. Most studies focus on probiotics derived from bacteria in the phylum *Bifidobacterium* and *Lactobacillus*, as they are known for their anti-inflammatory properties [109, 110]. Starting from animal studies, it is not difficult to observe that probiotics have a significant influence on the CNS [105, 111] (Fig. 3).

In a study with female mice that received an intraperitoneal injection of LPS, followed by treatment with a probiotic mixture containing *B. animalis*, *L. brevis*, and *Limosilactobacillus fermentum*, the probiotic treatment protected the animals from cognitive impairment and memory loss through anti-inflammatory and antioxidant mechanisms [112]. Furthermore, this effect is not only evident in wild-type animals; in another study with APP/PS1 mice, a transgenic model of AD, the treatment with extracellular vesicles from *L. paracasei* has upregulated neurotrophins, such as Brain-Derived Neurotrophic Factor (BDNF), NT3, NT4/5, TrkB, Mmp-2 and Mmp-9, and also was able to modify epigenetic mechanisms, alleviating the AD-like pathology [111]. In a similar study, APP/PS1 mice treated with *Lactobacillaceae* have shown improvement in cognitive function, decreased IL-6 levels, increased GSH-PX activity, and decreased MDA levels in the brain [109] (Fig. 3).

The reviewed preclinical studies show strong methodological convergence in the use of Bifidobacterium strains, primarily *B. breve* MCC1274, Bif11, BGN4, and *B. longum* BORI, administered primarily in murine models of AD, including APP knock-in, AppNL-G-F, and 5xFAD mice, as well as aging and depression-related behavioral models. These studies consistently investigate mechanisms within the gut–brain axis, such as metabolic alterations, neuroglial inflammatory responses, production of antioxidant metabolites, synaptic protection, and behavioral outcomes involving memory, cognition, and affective parameters. Across all models, probiotic administration produced measurable modulation of the cerebral microenvironment, involving antioxidant pathways, inflammatory regulation, synaptic plasticity, or amyloid/tau metabolism [113–123]. 

**Fig. 4:**
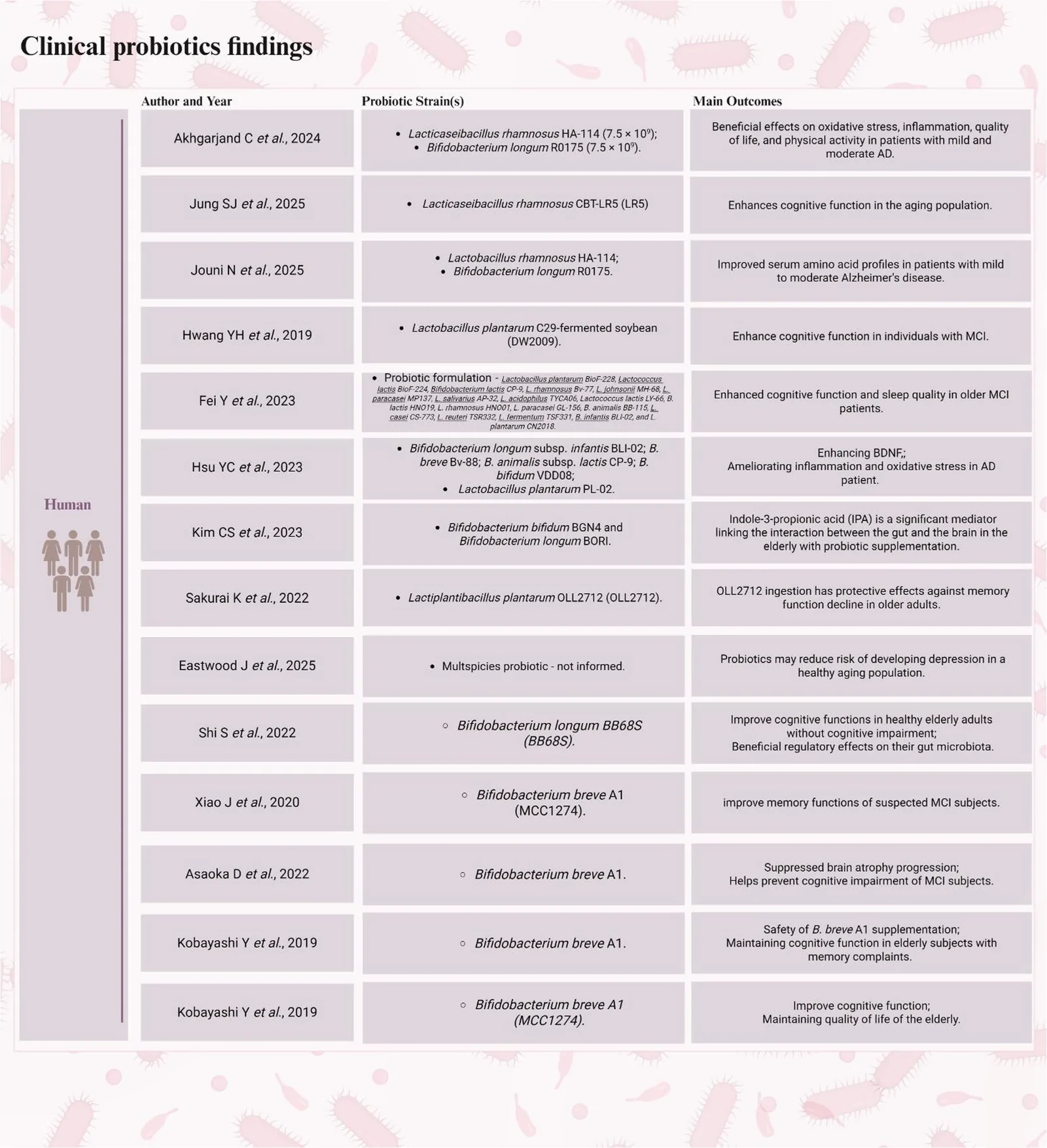
Clinical findings on the use of probiotics in human populations. The table presents clinical studies involving healthy older adults, MCI individuals , or AD patients. It describes the probiotic formulations used and their primary effects, including cognitive improvements, reductions in markers of inflammation and oxidative stress, enhanced quality of life, increased levels of neurotrophic factors, metabolic modulation, and a potential reduction in the risk of depression. Figure created with the assistance of BioRender (https://biorender.com*).*

The data go even further: humans have also shown positive effects on CNS health after probiotic treatment. *Bifidobacterium breve* A1 treatment for 16 weeks has improved the RBANS total score in older people, demonstrating the probiotic’s influence on human cognition [103]. With the same bacteria, after 24 weeks of treatment, older people showed improvement in subdomains of ADAS-Jcog and MMSE [124]. Another study, using *Bifidobacterium longum*, has also demonstrated that treatment with probiotics for just 8 weeks has significantly increased the scores of older people on the RBANS test [125] (Fig. 4).

Other clinical trials using various probiotic strains have yielded positive results following treatment; all have a 12-week treatment course. Unfortunately, a short number of studies had AD patients as the target group. Akhgarjand et al. (2024) used two different types of probiotics (*Lacticaseibacillus rhamnosus* HA-114 (7.5 × 109) or *Bifidobacterium longum* R0175 (7.5 × 109) and the probiotic supplementation for 12 weeks had beneficial effects on oxidative stress, inflammation, quality of life, and physical activity. Another study used the same probiotics as the first study (*Lacticaseibacillus rhamnosus* HA-114 or *Bifidobacterium longum* R0175), and found that the most significant improvements were consistently observed in the *B. longum* group. *B. longum* supplementation significantly improved serum amino acid profiles in patients with mild to moderate AD. Besides this, Hsu et al. (2023), using another strain (*Bifidobacterium longum subsp. infantis BLI-02*,* B. breve Bv-889*,* B. animalis subsp. lactis CP-9*,* B. bifidum VDD088*, and *Lactobacillus plantarum PL-02)* after 12 weeks of intervention, found that the individuals with AD who received the treatment demonstrated a 36% increase in serum BDNF, a reduction in IL-1β, and an increase in antioxidant superoxide dismutase (SOD) [85, 86, 104–128] (Fig. 4).

However, it is possible to summarize the main results obtained from clinical trials on the use of probiotics in individuals with AD or MCI. Probiotic supplementation, mainly with *Lactobacillus* and *Bifidobacterium*, improves cognitive function and reduces the release of inflammatory mediators and reactive oxygen species. In addition to the literature cited, it is worth highlighting that probiotics offer promising prospects as adjuvant therapeutic targets in early-stage AD and MCI [102, 104, 126–133] (Fig. 4).”

A few of these studies focused exclusively on cognitive scores and did not analyze the patients’ biochemical characteristics. However, other studies conducted biochemical analyses and found significant differences. In a study involving the Taiwanese population, elderly participants took a probiotic containing *Lactobacillus plantarum*,* Bifidobacterium bifidum*,* B. animalis*,* B. breve*, and *B. longum* for 12 weeks. It was observed that, although the individuals did not present a difference in their cognitive performance, there were increased levels of BDNF and SOD activity in the patients’ serum, as well as decreased levels of IL-1β, MDA, cortisol, and protein carbonylation content [126]. According to this study, elderly subjects treated with a probiotic composed of *B. bifidum* and *B. longum* for 12 weeks have been reported to exhibit a reduction in pro-inflammatory bacteria and increased serum levels of BDNF [134]. To reaffirm the correlation between the brain and gut microbiota, another study found increased BDNF levels in older people with mild cognitive impairment after 12 weeks of probiotic treatment [135] (Fig. 4).

**Fig. 5:**
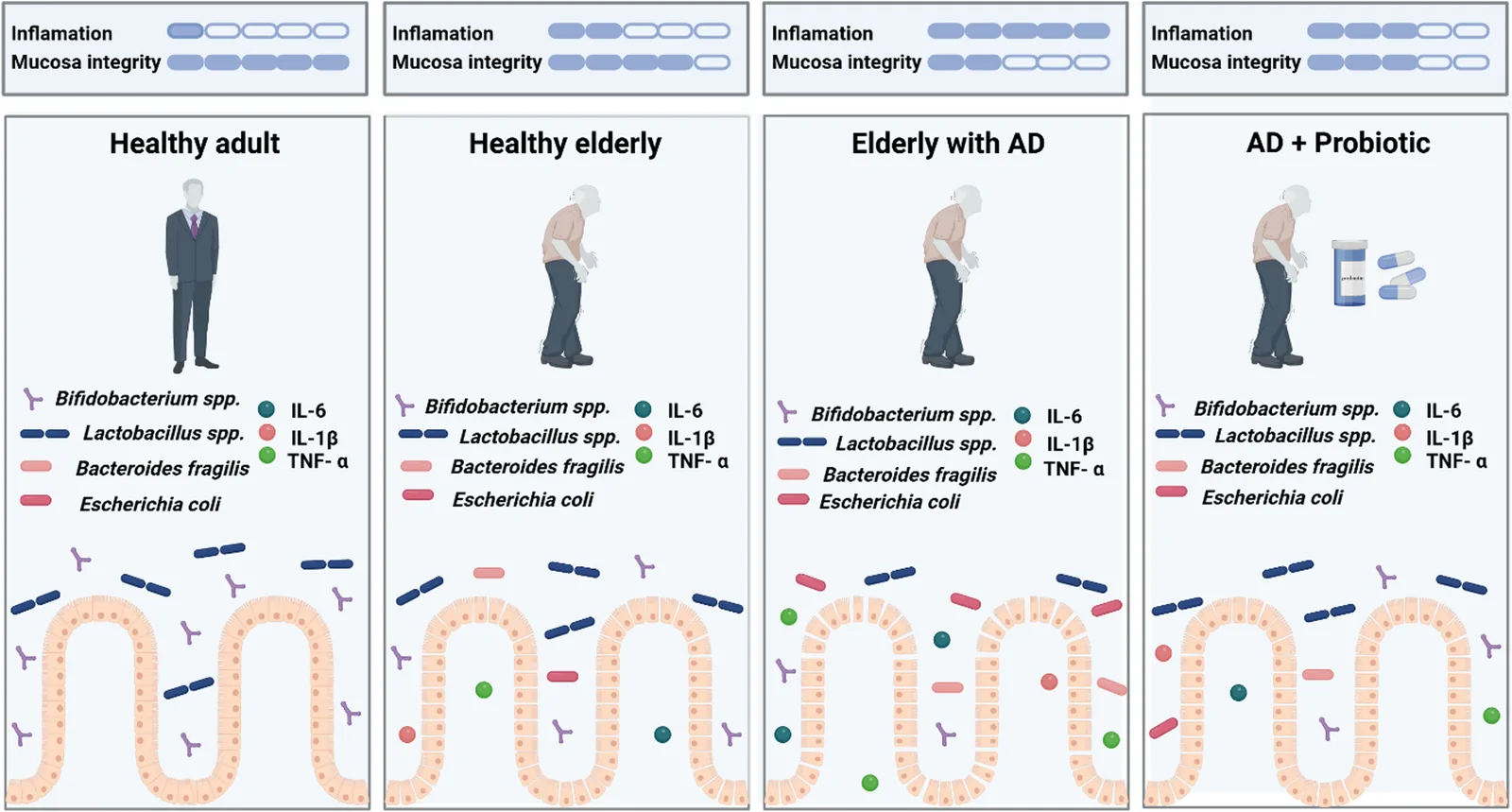
Changes in microbiota diversity throughout life decrease *Bifidobacterium* and *Lactobacillus* and increase intestinal permeability, pro-inflammatory cytokines, *Escherichia coli*, and *Bacteroides.* Probiotics offer microbiota benefits in older people with AD. In healthy adults, a balanced microbiota is observed, characterized by an abundance of *Bifidobacterium spp.* and *Lactobacillus spp.*, a low presence of potentially pathogenic bacteria such as *Bacteroides fragilis* and *Escherichia coli*, and low levels of pro-inflammatory cytokines (IL-6, IL-1β, and TNF-α), along with an intact intestinal mucosa. In healthy aging, there is a slight reduction in beneficial bacteria, an increase in potentially pathogenic bacteria, and a slight elevation of inflammatory markers, with a moderate decrease in mucosal integrity. In older adult individuals with AD, an imbalance in the intestinal microbiota is observed, characterized by a significant increase in *Escherichia coli* and *Bacteroides fragilis*, elevated levels of inflammatory cytokines, and a compromised intestinal barrier. Intervention with probiotics in elderly individuals with AD increases beneficial bacteria, reduces inflammatory cytokines, and improves intestinal mucosal integrity, suggesting a potential therapeutic benefit. Figure created with the assistance of BioRender (https://biorender.com*)*

Thus, in Fig. 5, we outline the benefits of probiotics in older individuals with AD, as reported in clinical studies. Treatment with probiotics has a positive effect on brain health and cognitive function, particularly those related to anti-inflammatory bacteria, such as *Bifidobacterium* and *Lactobacillus*. However, more studies are necessary to understand better the specific mechanisms by which bacteria increase BDNF and other neurotrophins, modulate microglia, and enhance cognitive performance.

**Fig. 6:**
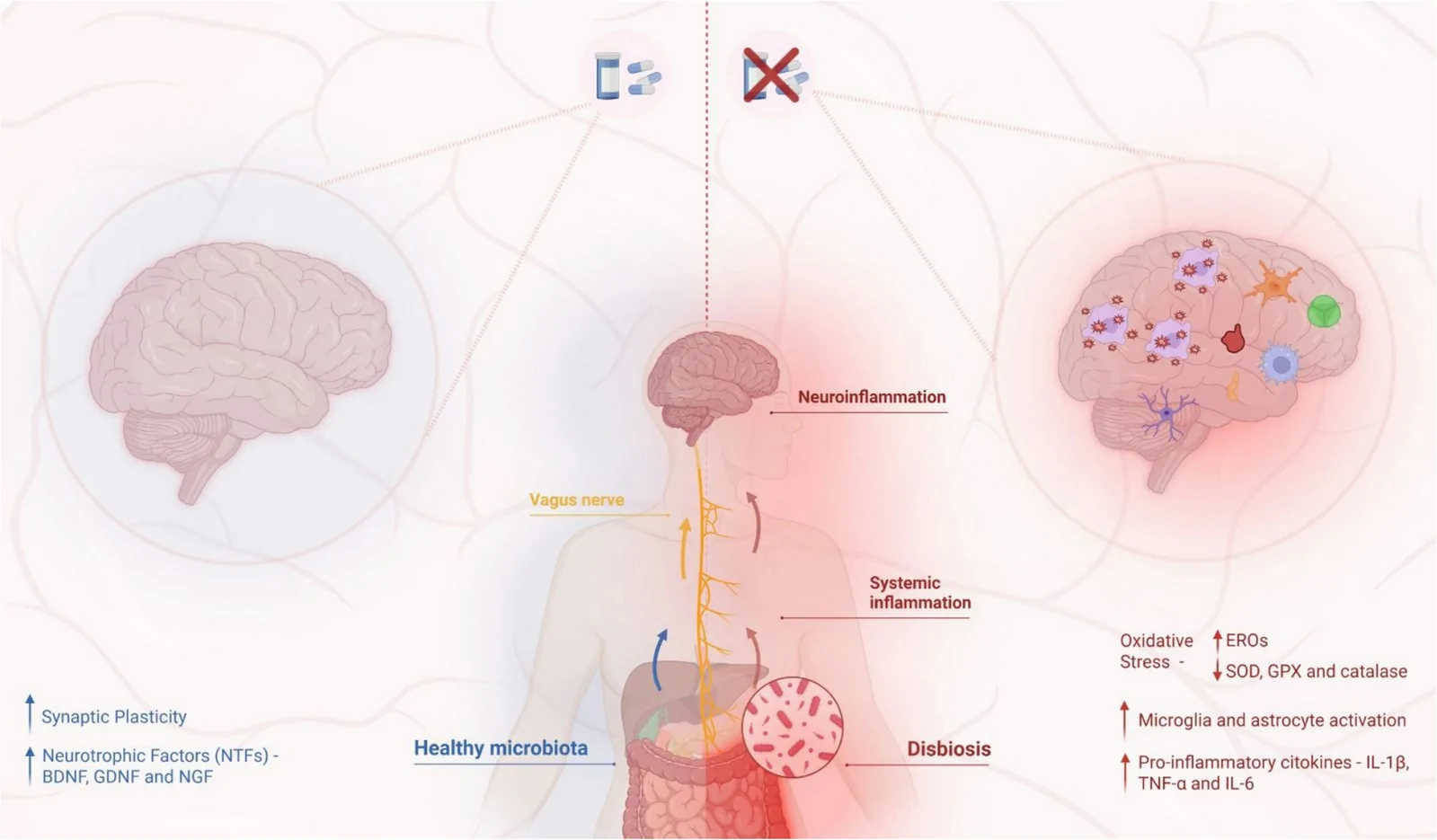
A proposed model integrating systemic inflammation, oxidative stress, and synaptic plasticity in the relationship between CNS and gut microbiota in healthy and neurodegenerative conditions. A healthy microbiota, supported by probiotic supplementation, promotes neuroprotection by increasing synaptic plasticity and elevating neurotrophic factors, including BDNF, GDNF, and NGF. In contrast, intestinal dysbiosis triggers systemic inflammation characterized by elevated levels of circulating cytokines (TNF-α, IL-6, IL-1β). These inflammatory mediators cross a compromised blood–brain barrier and activate microglia and perivascular macrophages, amplifying central neuroinflammation and reactive oxygen species production. Simultaneously, oxidative stress, marked by mitochondrial dysfunction and reduced antioxidant defenses such as SOD, GPX, and catalase, further enhances NF-κB and NLRP3 inflammasome signaling. Together, the inflammatory and oxidative pathways converge to impair neuronal function by destabilizing dendritic spines, reducing BDNF-mediated signaling, and decreasing long-term potentiation (LTP), ultimately compromising synaptic plasticity and cognitive reserve. Figure created with the assistance of BioRender (https://biorender.com*)*

Recent evidence increasingly challenges the reductionist view that dysbiosis “causes” neuroinflammation. Instead, the microbiota influences CNS homeostasis through context-dependent mechanisms. LPS translocation predominantly occurs when intestinal barrier function is compromised, a phenomenon that varies across individuals and disease states. In contrast, butyrate-producing bacteria modulate immune tolerance by epigenetically reprogramming regulatory T cells. The vagus nerve functions as a bidirectional communication pathway, transmitting both pro-inflammatory and anti-inflammatory signals depending on the microbial composition and local metabolite availability. In a healthy microbiota, increased synaptic plasticity and neurotrophic factors, such as BDNF, glial cell line-derived neurotrophic factor (GDNF), and nerve growth factor (NGF), help maintain CNS homeostasis. Conversely, in conditions of dysbiosis, increased microglial and astrocytic activation, accompanied by elevated levels of pro-inflammatory cytokines (IL-1β, TNF-α, and IL-6) and heightened production of reactive oxygen species (ROS), leads to reduced enzymatic antioxidant defenses. When these processes occur chronically and cyclically, they constitute the hallmark features of neuroinflammation [72, 118, 136, 137] (Fig. 6).

The convergence of cognitive benefits across preclinical and clinical models, coupled with consistent safety, establishes probiotics as a promising intervention for cognitive aging. However, critical translational gaps—notably the absence of microbiota characterization in humans, unvalidated Aβ effects, and methodological heterogeneity—prevent a definitive mechanistic understanding. Implementing the proposed harmonized framework with longitudinal microbiota analysis and APOE4 stratification would position future research to bridge the preclinical-clinical translational divide and enable evidence-based, personalized development of probiotic therapies for cognitive aging and AD prevention. Critically, most mechanistic evidence derives from rodent gnotobiotic models that fail to accurately recapitulate the complexity of the human microbiota or account for strain-level metabolic variation. In neurodegeneration, specifically, the link between microglial activation and dysbiosis, while statistically robust in cross-sectional studies, remains causally ambiguous. Longitudinal human data are sparse, and intervention trials (e.g., probiotic/prebiotic treatments) show modest or inconsistent effects. Disentangling authentic mechanistic relationships from epiphenomena will require integrative approaches combining untargeted metabolomics, single-cell transcriptomics, and carefully designed longitudinal cohorts [136, 137, 138].

## Conclusion

AD is complex and multifactorial. Gut dysbiosis has been consistently observed. The microbiota-gut-brain axis is a key element in understanding the mechanisms underlying neuroinflammation, offering promising avenues for developing new therapeutic strategies. Several factors, including infections, aging, exposure to toxins, systemic inflammation, and trauma, can trigger neuroinflammation in AD. This process is primarily mediated by microglia, leading to the increased production of pro-inflammatory cytokines, chemokines, prostaglandins, ROS, and nitric oxide, as well as the dysregulation of anti-inflammatory cytokines.

Gut dysbiosis has emerged as a likely contributor to this inflammatory cascade. In healthy adults, the gut microbiota is typically balanced, with a predominance of *Bifidobacterium spp.* and *Lactobacillus spp.*, low abundance of potentially pathogenic bacteria such as *Bacteroides fragilis* and *Escherichia coli*, and reduced levels of pro-inflammatory cytokines (IL-6, IL-1β, and TNF-α), alongside a preserved intestinal mucosal barrier.

During healthy aging, there is a moderate decline in beneficial bacteria, a rise in potentially pathogenic species, and a mild increase in systemic inflammatory markers, accompanied by partial impairment of mucosal integrity. In contrast, elderly individuals with AD exhibit a pronounced dysbiosis, characterized by a significant overgrowth of *Escherichia coli* and *Bacteroides fragilis*, elevated levels of pro-inflammatory cytokines, and substantial disruption of the intestinal barrier.

Probiotic supplementation has shown promise in this context, as it can help restore gut microbiota balance, reduce inflammatory cytokine levels, and enhance mucosal integrity. Additionally, probiotics may exert protective effects by modulating gut inflammation and regulating the gut-brain axis, representing a promising adjunctive approach for managing AD-related neuroinflammation.

Despite promising results, the evidence on the benefits of probiotics in patients with AD remains limited. The available literature presents a limited number of clinical trials and generally small sample sizes, which limits the statistical robustness and generalizability of the findings. Furthermore, there is considerable heterogeneity among studies, particularly regarding strains used, probiotic combinations, and doses, making it challenging to directly compare results and identify the most effective formulations. A relevant point is that the intervention time used is always 12 weeks. Another critical point to highlight is the lack of standardization in the outcomes evaluated, both clinical and biological, which contributes to the need for standardized protocols, multicenter studies, and larger sample population studies to confirm the potential therapeutic effects of probiotics in AD.

## Data Availability

No datasets were generated or analysed during the current study.
